# Mechanism of Ion Permeation in Mammalian Voltage-Gated Sodium Channels

**DOI:** 10.1371/journal.pone.0133000

**Published:** 2015-08-14

**Authors:** Somayeh Mahdavi, Serdar Kuyucak

**Affiliations:** School of Physics, University of Sydney, Sydney, New South Wales 2006, Australia; Zhejiang University, CHINA

## Abstract

Recent determination of the crystal structures of bacterial voltage-gated sodium (Na_V_) channels have raised hopes that modeling of the mammalian counterparts could soon be achieved. However, there are substantial differences between the pore domains of the bacterial and mammalian Na_V_ channels, which necessitates careful validation of mammalian homology models constructed from the bacterial Na_V_ structures. Such a validated homology model for the Na_V_1.4 channel was constructed recently using the extensive mutagenesis data available for binding of *μ*-conotoxins. Here we use this Na_V_1.4 model to study the ion permeation mechanism in mammalian Na_V_ channels. Linking of the DEKA residues in the selectivity filter with residues in the neighboring domains is found to be important for keeping the permeation pathway open. Molecular dynamics simulations and potential of mean force calculations reveal that there is a binding site for a Na^+^ ion just inside the DEKA locus, and 1–2 Na^+^ ions can occupy the vestibule near the EEDD ring. These sites are separated by a low free energy barrier, suggesting that inward conduction occurs when a Na^+^ ion in the vestibule goes over the free energy barrier and pushes the Na^+^ ion in the filter to the intracellular cavity, consistent with the classical knock-on mechanism. The Na_V_1.4 model also provides a good description of the observed Na^+^/K^+^ selectivity.

## Introduction

Voltage-gated sodium (Na_V_) channels enable fast and selective permeation of Na^+^ ions across membranes of excitable cells, and thereby play critical roles in electrical signaling in the nervous system, heart and muscles [1]. Dysfunctional Na_V_ channels are implicated in various disorders, which makes them potential drug targets for their treatment [2]. Thus a molecular-level understanding the operation of Na_V_ channels is an important problem in physiology with ramifications in medicine and pharmacology. For this reason, numerous experimental studies of Na_V_ channels have been conducted to decipher the structure-function relations [1, 3]. In particular, high-affinity toxin ligands have been used to probe the pore domain of Na_V_ and pinpoint the positions of functionally important residues [4–11]. However, in the absence of any crystal structures, it was difficult to uniquely interpret such data and obtain unambiguous structural information.

Recent determination of the crystal structures of bacterial Na_V_ channels [12–16] has the potential to ameliorate this situation. Several molecular dynamics (MD) simulations of the bacterial Na_V_ channels have been performed to study the binding sites of Na^+^ ions and their permeation mechanism [17–25]. Long MD simulations indicate that the selectivity filter (SF) is normally occupied by two Na^+^ ions in a waiting state, and conduction occurs when a third Na^+^ ion either knocks on them or passes by [23, 24]. To see whether a similar mechanism operates in mammalian Na_V_ channels, we need to construct reliable homology models from the bacterial counterparts. This is not as straightforward as in potassium channels, where the signature TVGYG sequence in the SF is conserved across the species. In contrast, the EEEE locus in the SF of the bacterial Na_V_Ab channel is replaced with the DEKA locus in mammalian Na_V_ channels, and an EEDD ring appears in the vestibule, three residues up from the DEKA locus. These are significant differences, and the insights obtained from the studies of the bacterial Na_V_ channels may not necessarily be transferable to the mammalian ones.

So far there has been a few attempts to construct homology models for the mammalian Na_V_1.4 channel using the Na_V_Ab crystal structure as a template [26–30]. The common choice of Na_V_1.4 in these studies is motivated by the fact that ample functional data are available for binding of *μ*-conotoxins to this channel, which could be used to constrain and validate the model. For example, our Na_V_1.4 model was validated using the data for binding of the *μ*-conotoxins, GIIIA, PIIIA, KIIIA, and BuIIIB [29, 30]. The binding modes obtained for all four *μ*-conotoxins were consistent with the available mutation data, and suggested existence of a common binding motif where the basic toxin residues formed ionic bonds with the residues of the EEDD ring. The proposed Na_V_1.4 model provides a reliable structure for the channel vestibule in general and the EEDD residues in particular, which are expected to play an important role in ion permeation. While the residues in the DEKA locus are not directly involved in binding of *μ*-conotoxins and could not be validated using the mutation data, their positions in the SF are rather well established, and there is much less uncertainty in regard to their modeling.

Here, we perform MD simulations to study the ion permeation properties of the proposed Na_V_1.4 model [29, 30]. Special attention is paid to the linking of the DEKA residues with those in the neighboring domains, which follow a different pattern from the corresponding EEEE residues in bacterial Na_V_ channels [12]. Potential of mean forces (PMFs) for a Na^+^ ion are constructed to locate the ion binding sites and elucidate the permeation mechanism. The Na^+^/K^+^ selectivity of the channel is explored by performing free energy perturbation (FEP) calculations at various sites. Our results indicate that the SF is occupied by a single Na^+^ ion, and conduction occurs via a knock-on mechanism when a second Na^+^ ion in the vestibule crosses a low free energy barrier and enters the SF.

## Methods

### Model of mammalian Na_V_1.4 channel

**Fig 1:**
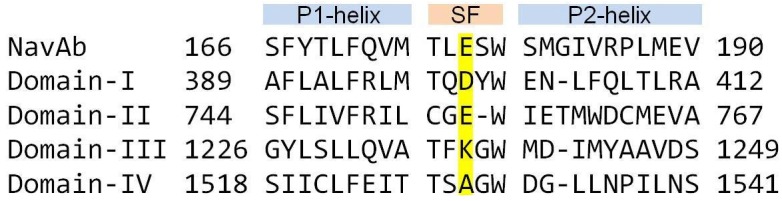
Alignment used in homology modeling of Na_V_1.4. The DEKA residues in the four domains forming the selectivity filter (SF) are highlighted.

The initial Na_V_1.4 model was constructed from the Na_V_Ab crystal structure (PDB ID: 3RVY) [12] using the alignment diagram in Fig 1. The 3-D model of the channel was created using Modeller [31] by threading the aligned Na_V_1.4 sequence for each domain on the corresponding domain of Na_V_Ab. The homology model was embedded in a lipid bilayer and solvated using the VMD software [32], and MD simulations of this system were performed to refine the model and to check its stability. Protocols developed for MD simulations of the gramicidin and potassium channels were used for this purpose [33, 34]. We refer to our previous work for further details of simulations and validation of the Na_V_1.4 model using the data from binding of *μ*-conotoxins [29, 30].

**Fig 2:**
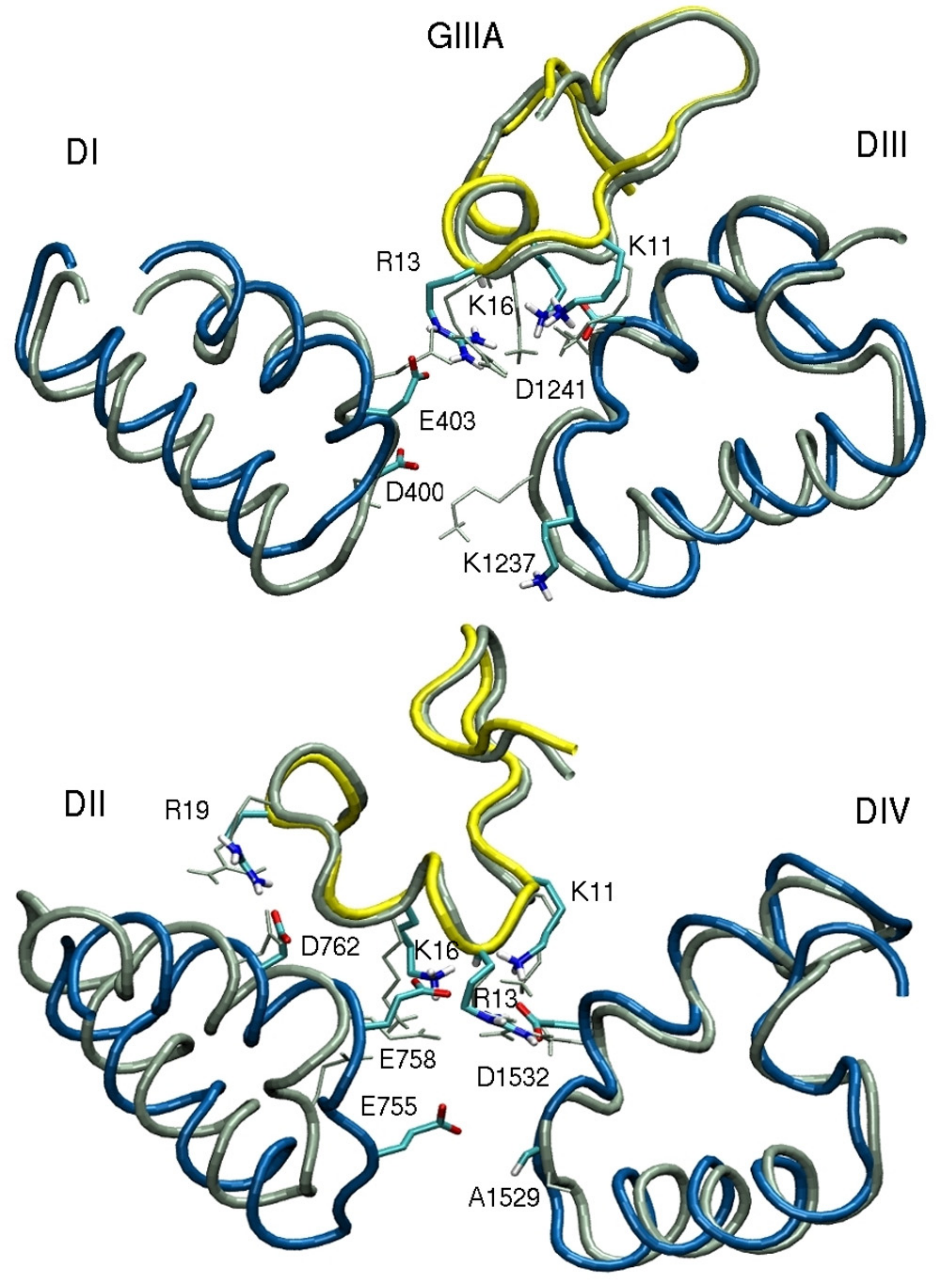
The binding modes of the Na_V_1.4–GIIIA complex in the new and previous Na_V_1.4 models. The old model is indicated with grey and the new one is shown in colour (Na_V_1.4 in blue and GIIIA in yellow). We note, in particular, the K1237 side chain in the old model, which protrudes to the pore center, blocking the channel.

The DEKA residues (D400, E755, K1237, and A1529) in the SF play a critical role in ion permeation, hence they need to be modeled correctly in order to obtain a working model of the Na_V_1.4 channel. To give an example, in some of the MD simulations of the Na_V_1.4 model based on 3RVY, the K1237 side chain was observed to form ionic bonds with the D400 side chain, blocking the pore (see Fig 2). To avoid such mishaps in modeling, we use the Na_V_Ab filter structure as a guide, where the side chains of the E177 residues corresponding to DEKA, make links with the backbone amides of S180 [12]. In the Na_V_1.4 model based on 3RVY, we have indeed identified potential partners for linking the side chains of the D400, E755, and K1237 residues. However, these links were not sustained in subsequent MD simulations of the model channel, leaving the side chains of the DEKA residues free. The 3RVY structure was obtained with the I217C mutation to trap the channel in a pre-open state with a closed pore. This mutation has had some effect on the structure of the SF [13], which may have prevented formation of stable inter-domain links in the Na_V_1.4 model based on 3RVY.

We have, therefore, constructed two more homology models for Na_V_1.4; one based on the wild type Na_V_Ab structure (PDP ID: 4EKW) [13], and the second based on the open-state Na_V_Ms structure (PDB ID: 4F4L) [15]. The vestibules in both models aligned well with that of the initial Na_V_1.4 model based on 3RVY. We have also checked that the binding mode of *μ*-conotoxin–GIIIA obtained in the earlier model [29] is reproduced in the new homology models. This is shown in Fig 2, where the Na_V_1.4–GIIIA complex obtained using the current Na_V_1.4 model based on 4EKW is compared to the previous one from ref. [29].

**Fig 3:**
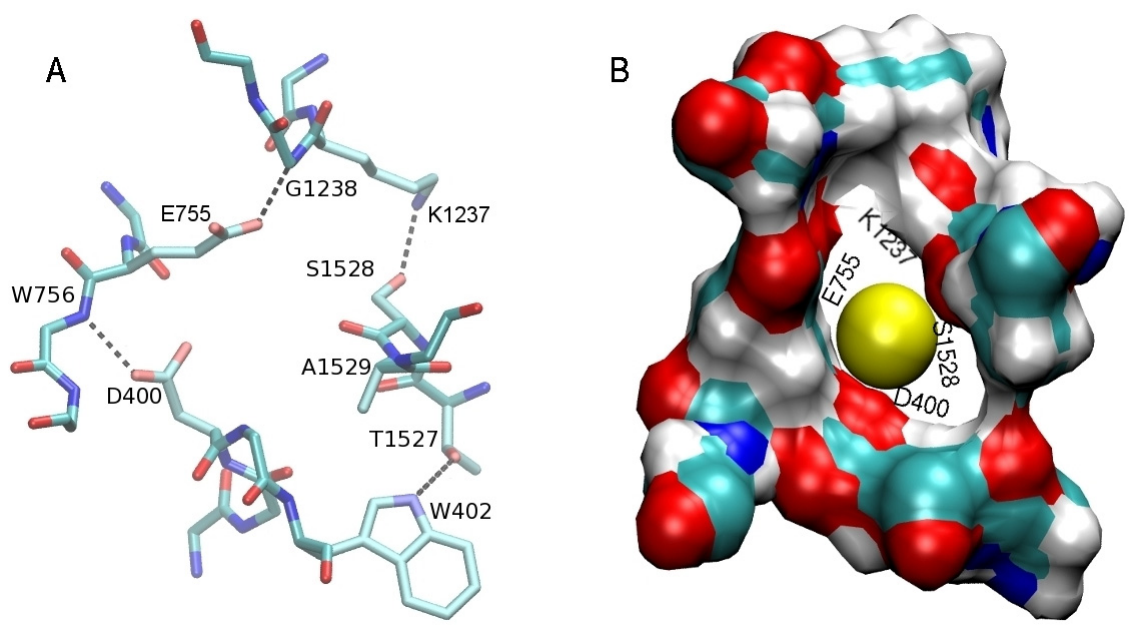
Structure of the SF. (A) Inter-domain linking interactions in SF of the Na_V_1.4 channel. (B) Space-filling diagram of the SF. The atoms facing the pore in the DEKA locus are indicated with labels. These are the side chain O atoms of D400 and E755, the backbone O atom of S1528, and the C_*α*_ atom of K1237. The O–O distances are; 5.9 Å for D-E, 5.5 Å for E-S, and 5.6 Å for S-D.

**Fig 4:**
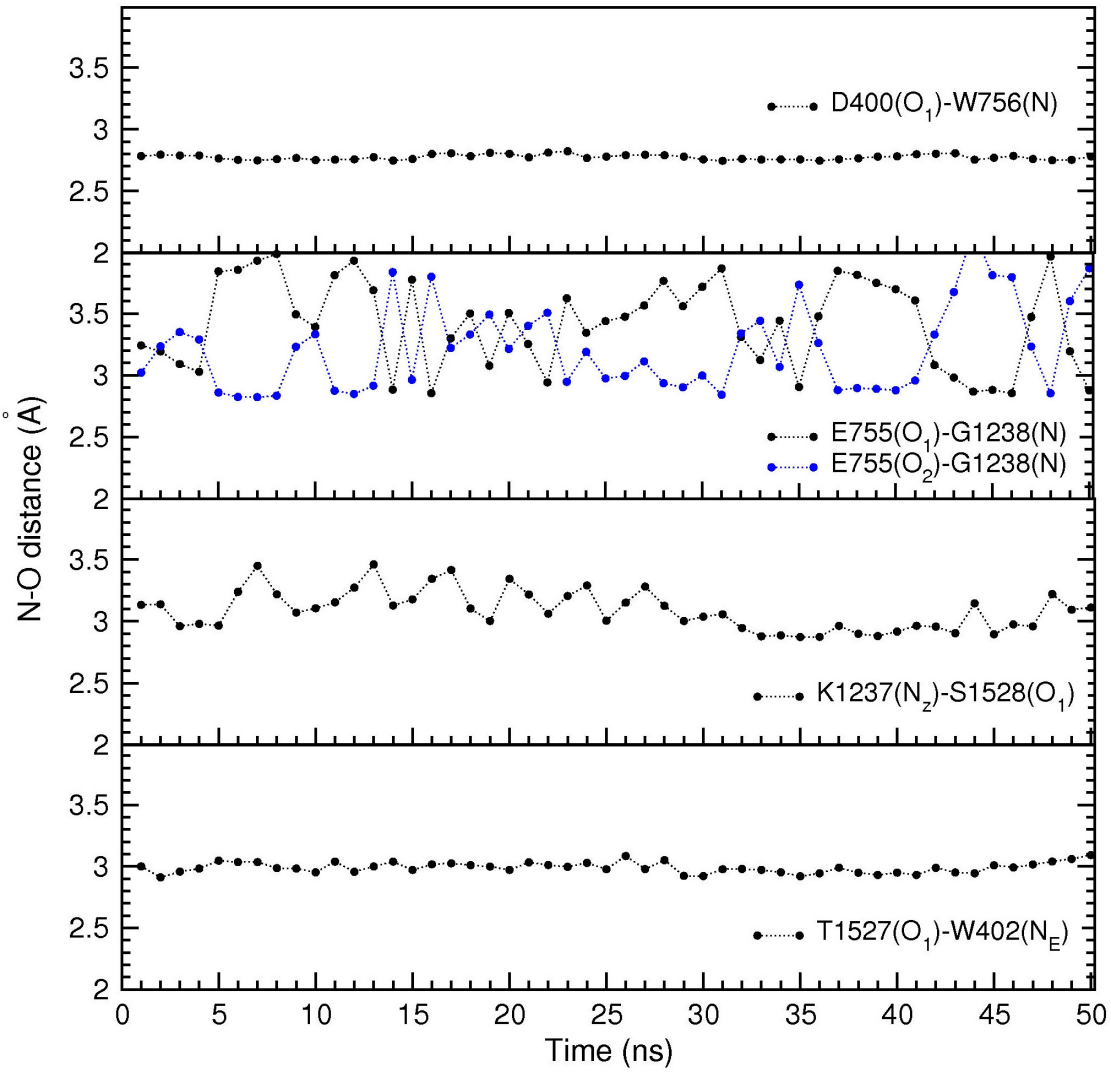
Time series of the N–O distances between the linked residues in the SF. The results are obtained from 50 ns of MD simulations, averaged over 1 ns blocks. The flipping of the side chain observed in E755 does not happen in D400 because the second oxygen in the side chain is involved in the coordination of the Na^+^ ion in the SF.

In both Na_V_1.4 models, unique inter-domain linking interactions are found between i) the side chain of D400 and the backbone amide of W756, ii) the side chain of E755 and the backbone amide of G1238, iii) the side chain of K1237 and the hydroxyl of S1528, and iv) the side chains of T1527 and W402 (Fig 3A). We note that this linking pattern is quite different from that observed in Na_V_Ab, where the E177 side chains are linked with the S180 amides which are three positions up from the EEEE locus. In the Na_V_1.4 model, the D400 and E755 side chains are linked with the backbone amide of residues that are only one position up from the DEKA locus. In the case of the K1237 side chain, the link is made with the side chain of a residue that is one position down from the DEKA locus. The last link between T1527 and W402 makes up for the missing link from A1529 and helps to complete the ring around the filter. A space filling diagram of the resulting SF is shown in Fig 3B. The O–O distances between the O atoms facing the pore are quoted in the caption, which indicate that the SF is wide enough for passage of a Na^+^ ion. We have checked the stability of these inter-domain links in MD simulations of the Na_V_1.4 model based on the 4EKW structure. As shown in Fig 4, the N–O distances between the linked residues have remained stable during 50 ns of MD simulations. Therefore, the Na_V_1.4 model based on 4EKW has been used in all subsequent studies of ion permeation in this work. We have monitored these inter-domain links during the umbrella sampling and other MD simulations of the Na_V_1.4 model, and confirmed their stability over much longer time scales (over 1 *μ*s in total),

### MD simulations and free energy calculations

MD simulations are performed using NAMD (version 2.9) [35] with the CHARMM36 force field [36]. An NpT ensemble is used with periodic boundary conditions. Pressure is kept at 1 atm and temperature at 300 K using Langevin coupling with damping coefficients of 5 ps^−1^. Lennard-Jones interactions are switched off smoothly within a distance of 10–13.5 Å. Electrostatic interactions are computed using the particle-mesh Ewald algorithm. The channel protein has a net charge of −8*e*, which is neutralized by adding 8 extra Na^+^ ions to the 100 mM NaCl solution. A time step of 2 fs is used, and the trajectory data is saved at 1 ps intervals. In umbrella sampling simulations, the reaction coordinate is recorded at every time step.

The PMFs of a Na^+^ ion in the Na_V_1.4 model based on 4EKW are constructed using umbrella sampling MD simulations. Because application of the method to the gramicidin and potassium channels has been described in detail before [37, 38], we give only a brief description here. The reaction coordinate is taken as the distance of the ion from the center of mass of the Na_V_1.4 protein along the channel axis (*z*-axis). Two PMFs are constructed; one with no other Na^+^ ions in the channel and a second one with a Na^+^ ion initially resident in the SF. In the first PMF, 40 umbrella windows are created along the channel axis at 0.5 Å intervals. Starting from the equilibrated position of the Na^+^ ion in the SF, the ion is pulled in steered MD simulations with a speed *v* = 5 Å/ns for 0.1 ns to create the successive windows. Each window is equilibrated for 0.1 ns before proceeding with the generation of the next window. A force constant of *k* = 10 kcal/mol/Å^2^ is used in both steered MD and umbrella sampling simulations. Extra windows are introduced when the overlap of distributions between the neighboring windows drops below 5% to avoid numerical instabilities in the construction of the PMF (e.g., between the windows 9–10, 17–18, 18–19, 21–22 and 26–27). The PMF is extended in the bulk region until it becomes flat, which signals the bulk conditions. The reaction coordinates collected from the simulations are unbiased and combined using the weighted histogram analysis method [39]. Umbrella sampling simulations are continued until the convergence of the PMF is assured from block data analysis of the PMF data.

A similar procedure is followed for the second PMF. The starting configuration is similar to that in the first PMF except a second Na^+^ ion is placed on the channel axis outside the vestibule. The second Na^+^ ion is pulled inside the pore via steered MD, creating 40 umbrella windows along the channel axis. No biasing potentials are applied to the first Na^+^ ion in the filter during the umbrella sampling MD simulations.

The Na^+^/K^+^ selectivity of the channel is investigated by performing FEP calculations at several sites. The Na^+^/K^+^ selectivity free energy is given by the difference of the free energy change in a site and in bulk

$$\Delta \Delta G_{\text{sel}}\left({\text{Na}}^{+}\to K^{+}\right)=\Delta G_{\text{site}}\left({\text{Na}}^{+}\to K^{+}\right)-\Delta G_{\text{bulk}}\left({\text{Na}}^{+}\to K^{+}\right)$$ (1)

To minimize systematic errors, we perform the FEP calculations at the site and bulk simultaneously. That is, in a forward FEP calculation, a Na^+^ ion is transformed to a K^+^ ion at the site while a K^+^ ion in bulk is transformed simultaneously to a Na^+^ ion. To check for hysteresis effects, we have also performed backward FEP calculations. The final FEP result is obtained from the average of the forward and the negative of the backward results. FEP calculations are performed using 66 exponentially-spaced windows [40]. Each window is equilibrated for 50 ps and followed by 50 ps production run. The errors are estimated using the ParseFEP plugin in NAMD.

## Results and Discussion

### MD simulations of the Na_V_1.4 channel

**Fig 5:**
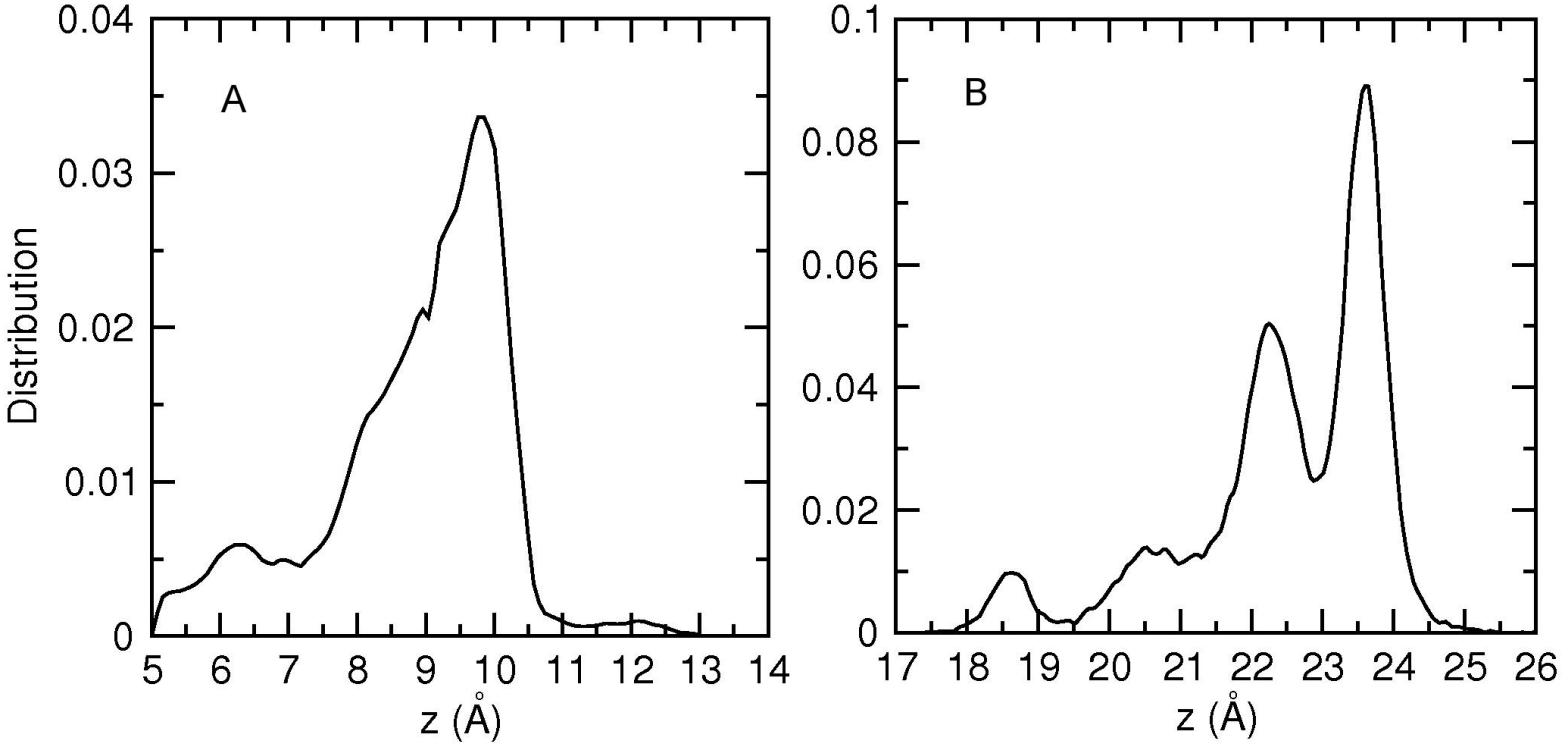
Distribution of the Na^+^ ions in the pore. A) Distribution of the *z*-coordinate of the Na^+^ ion in the SF obtained from a 50 ns MD simulation. B) Distribution of the *z*-coordinates of Na^+^ ions in the vestibule obtained from a 125 ns MD simulation.

**Fig 6:**
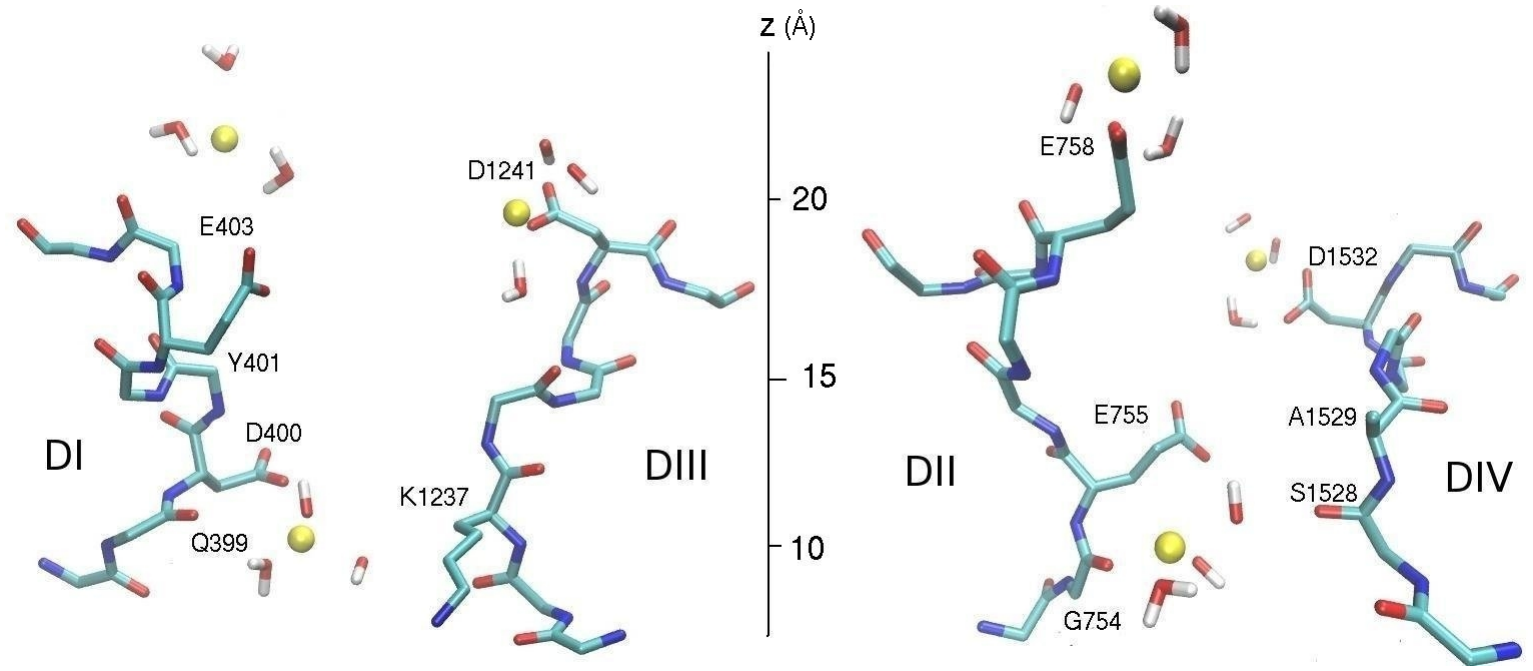
Snapshot of the pore domain of the Na_V_1.4 model depicting Na^+^ ions. One Na^+^ ion is in the SF at *z* = 9.7 Å, and two Na^+^ ions are in the vestibule at *z* = 21.6 and 23.5 Å. The two ions in the vestibule are in off-axis positions and separated by about 11 Å. The domains DI and DIII (left) and DII and DIV (right) are shown separately for clarity.

To locate potential binding sites of Na^+^ ions in the Na_V_1.4 model, we have first performed several unbiased MD simulations with varying number of Na^+^ ions in the channel (the bulk NaCl concentration is kept at 0.3 M). When a Na^+^ ion is placed near the DEKA locus, it is observed to move further in to the SF within a few ns and then remain in a well-defined binding site during the rest of the simulations for 50 ns. The distribution of the *z*-coordinate of the Na^+^ ion from one of the MD simulations is shown in Fig 5A. The ion makes occasional excursions to the cavity but remains in the SF for most of the time. A Gaussian fit to the data points in the peak region indicates that the binding site in the SF is around *z* = 9.7 Å, slightly below the DEKA locus (the center of mass of the channel protein is at *z* = 10.0 Å). At this site, the Na^+^ ion is coordinated by the oxygens of the side chain of D400, the carbonyl of G754, and 3–4 water molecules (see Fig 6 for snapshots). The SF can bind only one Na^+^ ion. When a second Na^+^ ion is placed in the vicinity of the first one in the SF, the first ion quickly moves in to the cavity and remains there. This is consistent with electronegativity arguments based on the total charge of −*e* at the DEKA locus, which would be neutralized by the +*e* charge of a Na^+^ ion.

We note that the linking of the side chain of K1237 with the side chain of S1528 is critical for the formation of the SF binding site as it keeps the positive charge of lysine away from this site. To see what happens if this linking is removed, we have performed MD simulations of the S1528A analog of the channel with a Na^+^ ion at the SF binding site for 25 ns. The K1237 side chain is seen to be released from its off-axis position, getting closer to the Na^+^ ion. This results in destabilization of the Na^+^ ion, which is pushed towards the vestibule. It will be interesting to check the effect of the S1528A mutation on the conductance of the channel. The S1528 side chain is not involved in the coordination of a Na^+^ ion in the SF, and the other inter-domain links involving D400, E755, and T1527 are not affected by this mutation. Thus any reduction in the conductance due to the S1528A mutation could provide direct evidence for the proposed K1237–S1528 inter-domain linking.

We have next explored binding sites in the vestibule by placing a Na^+^ ion near the EEDD ring while there is a Na^+^ ion resident in the SF. Although the Na^+^ ion remains in the vestibule during the course of the MD simulations, there is no unique binding site as in the case of the SF. In addition, another Na^+^ ion is observed to enter the vestibule and remain there for substantial times, coordinated by the side chain of E758 or, to a lesser extent, E403. One of the common positions of the two Na^+^ ions in the vestibule is shown in Fig 6, where the inner Na^+^ ion is coordinated by the side chains of D1241 and D1532, and four water molecules while the outer Na^+^ ion is coordinated by the side chain of E758 and four water molecules. The distribution of the *z*-coordinate of the Na^+^ ions in the vestibule (Fig 5B) provides a more quantitative picture for the ion positions. From the integral of the distribution curve, the average number of Na^+^ ions in the vestibule is obtained as 1.6. One Na^+^ ion is always resident in the vestibule, coordinated by one or two of the EEDD side chains. When there is a second Na^+^ ion in the vestibule, it is localized around *z* = 23.6 Å (the sharp peak in Fig 5B). Its further entry into the vestibule is presumably prevented by the Coulomb repulsion of the first Na^+^ ion in the vestibule. Another interesting observation from Fig 5 is that the region *z* = 13–18 Å is not occupied by Na^+^ ions at all. This hints to the presence of a free energy barrier at the entrance of the SF, which is expected to play an important role in permeation of Na^+^ ions.

### PMF calculations

**Fig 7:**
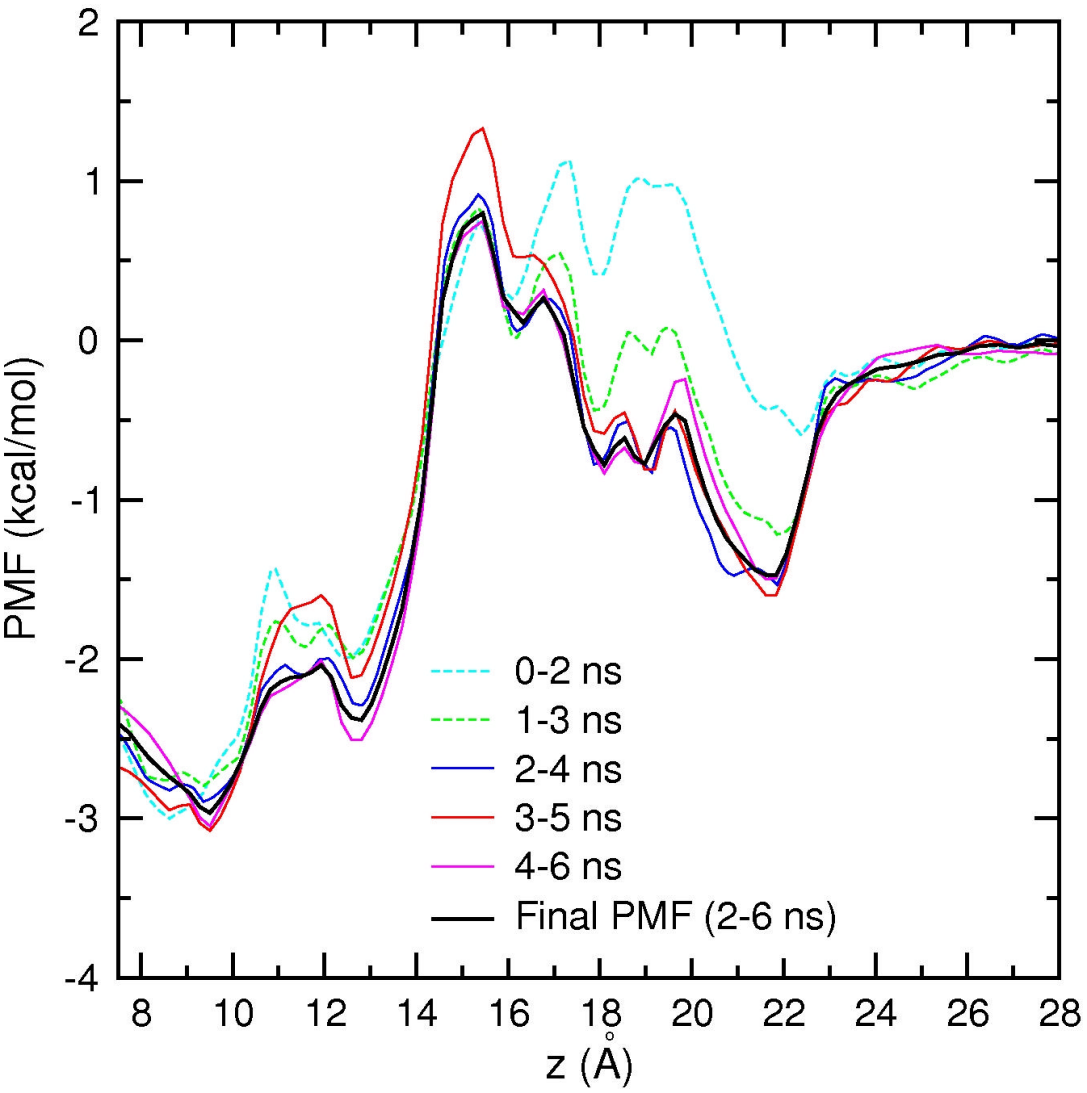
PMF of a Na^+^ ion in the Na_V_1.4 channel with no resident Na^+^ ions. Block data analysis using 2 ns blocks indicates that the PMF converges after 2 ns of equilibration. Therefore the first 2 ns is excluded, and the final PMF is obtained from 2–6 ns of the data.

Unbiased MD simulations of the Na_V_1.4 channel suggest that the permeation mechanism involves two Na^+^ ions separated by a free energy barrier between the SF and the vestibule. To elucidate the permeation mechanism further and provide a more quantitative description of ion permeation, we construct PMFs for a Na^+^ ion along the channel axis. The first PMF in Fig 7 shows the free energy profile of a Na^+^ ion when there are no other ions in the pore. Each umbrella window is simulated for 6 ns to ensure convergence of the PMF. Block data analysis of the PMF data in Fig 7 indicates that a converged PMF is obtained after 2 ns of equilibration, therefore the final PMF (shown with a black curve) is constructed from 2–6 ns of the data. The PMF shows that there is a shallow binding pocket in the vestibule at around *z* = 22 Å with a depth −1.5 kcal/mol, and another binding site in the SF at *z* = 9.5 Å with a depth −3 kcal/mol. Thus a Na^+^ ion in the vestibule is expected to cross a low free energy barrier of 2 kcal/mol and fall in to the free energy minimum at the SF. The free energy barrier is centered at *z* = 15.5 Å, and the binding site in the SF is at *z* = 9.5 Å, which are consistent with the results of the unbiased MD simulations of Na^+^ ions.

**Fig 8:**
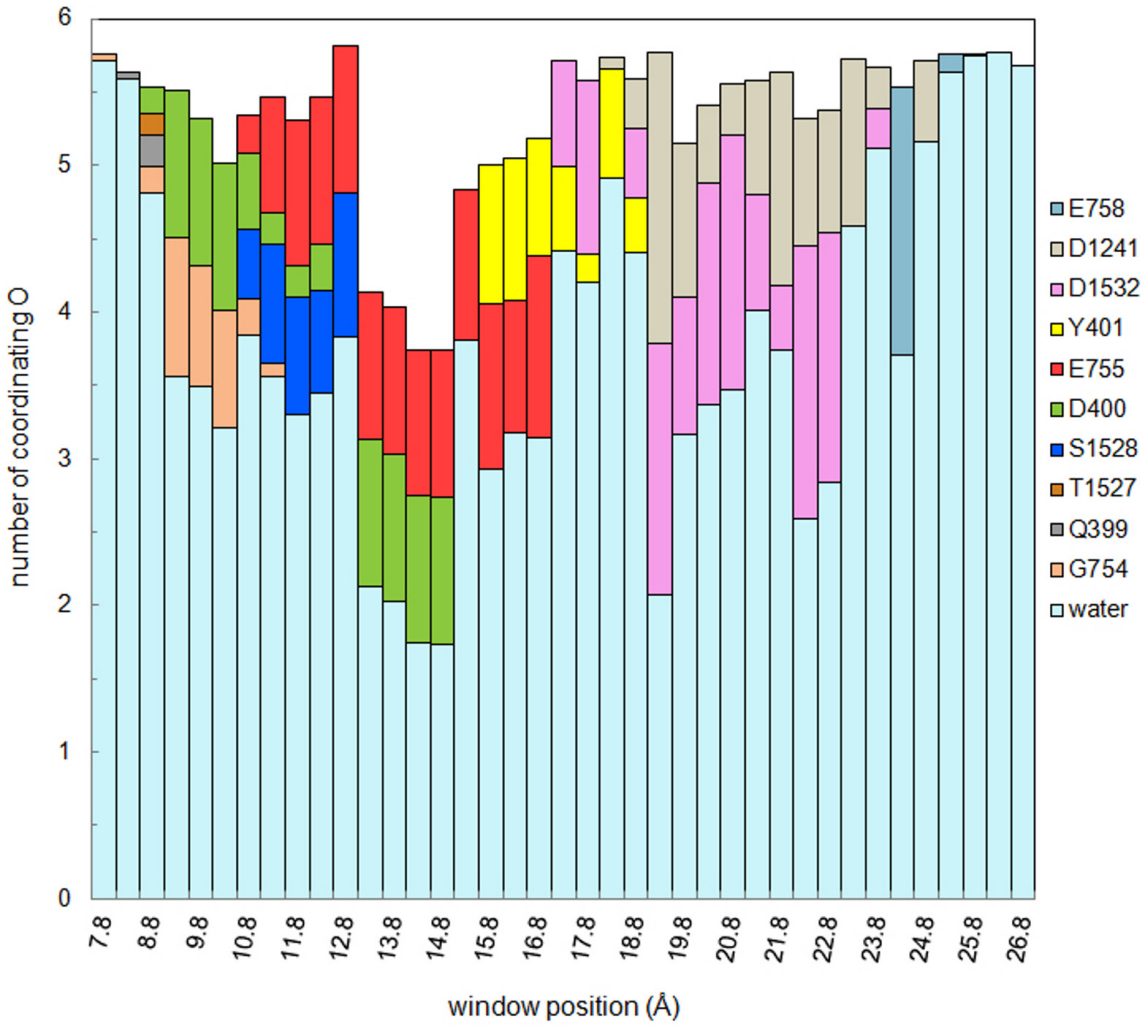
Average number of oxygen atoms coordinating the Na^+^ ion in the Na_V_1.4 pore. The results are obtained using 2–6 ns of the PMF data from each umbrella sampling window. Contribution from each residue and water molecules are shown with different colors.

To get a better understanding of the main features of the PMF in Fig 7, we have analyzed how the Na^+^ ion is coordinated in each umbrella window. In Fig 8, we show the average number of oxygens from individual protein residues and water molecules contributing to the coordination of the Na^+^ ion. A cut off radius of 3 Å is used for the Na–O distance. The sharp rise of the PMF between *z* = 13–15 Å is seen to be caused by the sudden drop in the number of water molecules coordinating the ion from about four to two. This region is the narrowest part of the SF, and there is not enough room for a Na^+^ ion with more than two hydration waters. Only two oxygens from the side chains of D400 and E755 from the DEKA locus contribute to the coordination of the Na^+^ ion, which is not sufficient to replace the missing water oxygens, and leads to the sharp rise in the PMF. After the free energy barrier (*z* > 18 Å), the Na^+^ ion is well coordinated by close to six oxygens from water molecules and protein. In particular, the side chains of D1241 and D1532 from the EEDD ring are seen to be involved in the creation of the shallow binding pocket around *z* = 22 Å. The loose nature of the binding positions in the vestibule, the high number of water molecules coordinating the Na^+^ ion, and the relatively low contribution from protein oxygens—which keeps changing—suggest that the Na^+^ ions are stabilized mainly by electrostatic interactions in the vestibule.

**Fig 9:**
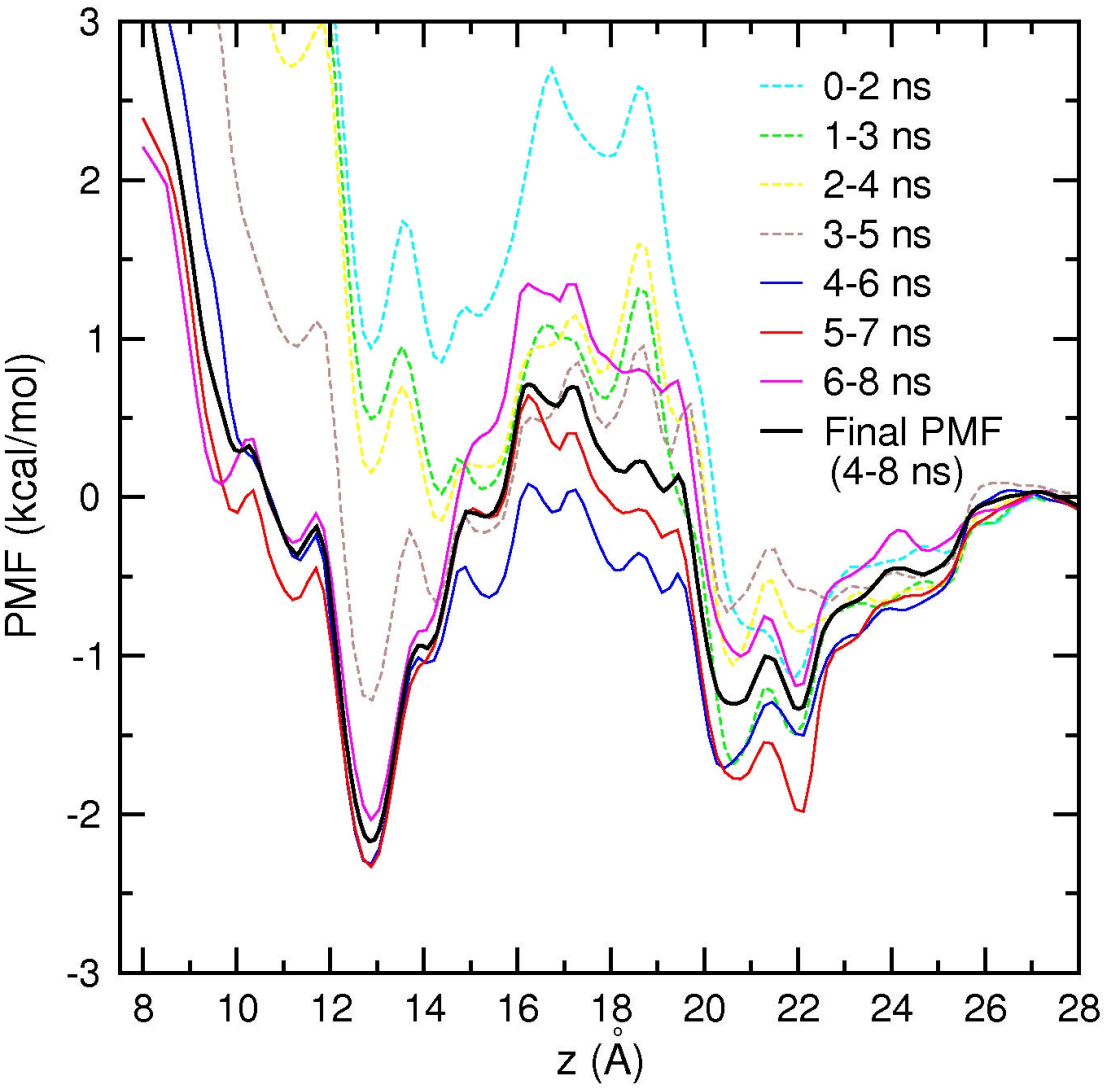
PMF of a Na^+^ ion in the Na_V_1.4 channel with a Na^+^ ion resident in the SF. Block data analysis using 2 ns blocks indicates that the PMF converges after 4 ns of equilibration. Therefore the final PMF is obtained from 4–8 ns of the data.

The unbiased MD simulations indicate that the vestibule can hold at least one Na^+^ ion while there is a Na^+^ ion in the SF. To explore the role of a Na^+^ ion in the vestibule in the permeation mechanism, we construct a second PMF by bringing a Na^+^ ion from the bulk region to the SF, while there is an unrestrained Na^+^ ion in the SF (Fig 9). Because the Na^+^ ion in the SF is free, convergence of the PMF is slower. From block data analysis of the data, we exclude the first 4 ns of data and construct the final PMF from 4–8 ns of data. Comparison of the PMF in Fig 9 with that in Fig 7 shows that the main features of the single-ion PMF are preserved in the presence of a Na^+^ ion in the SF. There is a shallow binding pocket of depth −1.5 kcal/mol in the vestibule, which will attract a second Na^+^ ion. This ion faces an energy barrier of 2 kcal/mol to reach the SF, where it has a stable minimum at *z* = 13 Å. During this process, the first Na^+^ ion in the SF is pushed to *z* = 5 Å, where it stays in a semi-stable position. This happens because the channel is in a closed state, and the ion is trapped in the inner cavity. Thus the sharp rise in the PMF for *z* < 13 Å, and shifting of the SF binding site from *z* = 9.5 Å in Fig 7 to *z* = 13 Å in Fig 9 are caused by the Coulomb repulsion of the Na^+^ ion in the cavity. In an open channel, the Na^+^ ion in the cavity would be ejected from the channel, and the binding site would revert back to *z* = 9.5 Å as found in the single-ion PMF in Fig 7.

**Fig 10:**
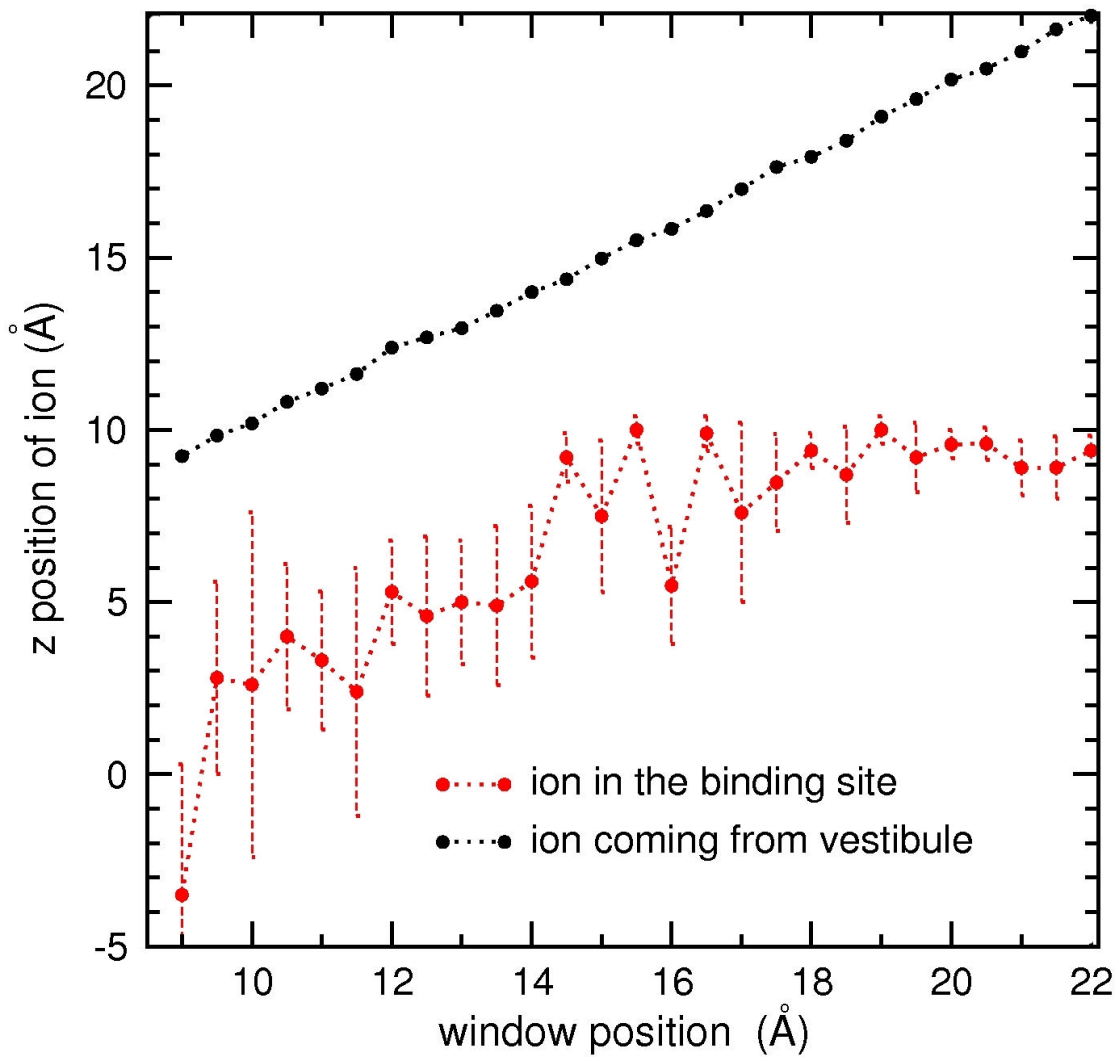
Correlated motion of two Na^+^ ions in the pore depicting the knock-on mechanism. Changes in the average *z*-position and fluctuations of the Na^+^ ion in the SF binding site (red dots and dotted lines) while a second Na^+^ ion is moved from the vestibule to the SF in umbrella sampling simulations (the *z*-coordinate is indicated by black dots).

In order to illustrate the correlated motion of the two Na^+^ ions in the SF and the vestibule, we show their average positions in each umbrella window in Fig 10. As the outer Na^+^ ion approaches the SF, the inner Na^+^ ion mostly remains in the SF but makes increasingly more frequent excursions to the cavity. When the outer Na^+^ ion reaches *z* = 14 Å, corresponding to the downward slide in the PMF (Fig 9), the Coulomb repulsion is sufficient to dislodge the inner Na^+^ ion from the SF, which moves in to the cavity. Thus the critical event in the permeation cycle is a Na^+^ ion in the vestibule crossing over the free energy barrier at the entrance of the SF. Once over this barrier, the ion moves towards the SF under an attractive mean force, which triggers the classical knock-on mechanism, mediated by the repulsive Coulomb force.

Because the model Na_V_1.4 channel is in a closed state, it is not possible to estimate its conductance and compare with experiments. Nevertheless, assuming that the rate-limiting step occurs in the SF, we can infer the permeation properties of the model channel by comparing the PMF results with those obtained from the potassium channels [38, 41]. The PMFs in Figs 7 and 9 indicate presence of a single free energy barrier in the SF and vestibule regions, which is lower than the typical free energy barriers found in the potassium channels. Thus it is expected that the model Na_V_1.4 channel in the open state should be able to conduct Na^+^ ions near-diffusion rates as found in the potassium channels [42].

The effect of the mutation of the DEKA and EEDD residues on ion permeation in Na_V_1 channels has been studied in several papers previously [5, 43–46]. While detailed tests of the effect of these mutations is beyond the scope of this work, we can nevertheless check their consistency with the model results. Mutation of the E403 and E758 residues to glutamine has the largest effect on the conductance, reducing it by 97% and 80%, respectively, while the mutation of D1241 and D1532 to asparagine has much less effect on the conductance, reducing it by a few percent and 43%, respectively [5]. Mutation of the EEDD residues to cysteine has a comparatively lower impact on the conductance, suggesting that these residues are directly involved in ion coordination, rather than just providing an electrostatic attraction for ions [43]. In unbiased MD simulations, we have observed that E403, and with more probability E758, provide initial anchoring points for Na^+^ ions entering the vestibule, providing two coordinating oxygens each. In contrast, D1241 and D1532 provide typically one coordinating oxygen each, with D1532 making a larger contribution than D1241 (see Fig 8). Thus, consistent with experimental observations, we expect neutralization of E758 to reduce the conductance most, followed by E403, D1532 and D1241.

In the DEKA locus, alanine mutation of D400 and E755 reduces the conductance by 20% and 30%, respectively while K1237A increases the conductance by almost four fold [45]. Neutralization of the positive charge in the SF will clearly create a deeper binding site in the SF, which should help to attract Na^+^ ions from the vestibule and thereby boost the conductance. The D400 and E755 side chains contribute an oxygen each to a Na^+^ ion in the SF (Fig 8), so their mutation to alanine is expected to reduce the conductance. To understand why the reduction is fairly small, we have performed separate MD simulations for the D400A and E755A analogs of the channel with a Na^+^ ion in the SF for 20 ns. The space emptied by the side chain of D400 or E755 is observed to be occupied by a water molecule, which substitutes for the missing protein oxygen, and hence reduces the energetic penalty arising from the alanine mutation.

### Ion selectivity

Because of the lack of evolutionary pressure, ion selectivity in Na_V_ channels is not as well-developed as in K_V_ channels. The observed Na^+^/K^+^ permeation ratios in Na_V_ channels are in the range 10–30, which translates to selectivity free energies 1.4–2.0 kcal/mol [45–47]. We have performed FEP calculations at several sites in the Na_V_1.4 model to investigate its Na^+^/K^+^ selectivity. The sites in Table 1 are chosen according to the ion distribution and PMF results, with *z* = 31 Å representing bulk. The last site at *z* = 8.9 Å is the closest location of the Na^+^ ion to the K1237 side chain where it remains in a semi-stable equilibrium. This site is chosen because the K1237A mutation has been observed to abolish the Na^+^/K^+^ selectivity [45].

**Table 1:** Na^+^/K^+^ selectivity free energies at various ion positions. The ΔΔ*G*_sel_(Na^+^ → K^+^) values are obtained from FEP calculations using Eq 1 (in units of kcal/mol). FW and BW refer to the forward and backward FEP calculations, respectively. The final result in the last column is obtained from the average of the FW and −BW. Star in the last two rows indicate that the FEP calculations are performed for the K1237A analog of the Na_V_1.4 model.

z (Å)	FW	−BW	Average
31	0.1 ± 0.1	0.1 ± 0.1	0.1 ± 0.1
21	0.6 ± 0.1	0.4 ± 0.1	0.5 ± 0.1
13	0.9 ± 0.2	1.0 ± 0.2	1.0 ± 0.3
9.7	0.9 ± 0.1	0.9 ± 0.1	0.9 ± 0.1
8.9	1.5 ± 0.1	1.3 ± 0.1	1.4 ± 0.1
9.7*	0.6 ± 0.1	0.4 ± 0.1	0.5 ± 0.1
8.9*	−0.1 ± 0.1	−0.2 ± 0.1	−0.1 ± 0.1

The FEP results obtained from the forward and backward calculations, and their average are presented in Table 1. Comparison of the forward and backward FEP results show that hysteresis effects are minimal. As expected, there is a marginal Na^+^/K^+^ selectivity in the wider vestibule, and appreciable selectivity occurs in the narrow SF. Of particular interest is the site at *z* = 8.9 Å, where the Na^+^/K^+^ selectivity is the highest (1.4 kcal/mol), which is also commensurate with the experimental results. To confirm the role of the K1237 residue in the ion selectivity of the Na_V_1.4 model, we have repeated the FEP calculations in the SF using the K1237A analog of the Na_V_1.4 model (the last two rows in Table 1). The selectivity free energy is reduced to 0.5 kcal/mol at *z* = 9.7 Å, and the Na^+^/K^+^ selectivity is completely abolished at *z* = 8.9 Å (Table 1, last row), consistent with the observations [45]. A similar study of the Na^+^/K^+^ selectivity was performed previously on a Na_V_1 model, which did not have the inter-domain links in the DEKA locus as proposed here [48]. As one would expect, their results for the selectivity free energy widely varied depending on the location of the K1237 side chain. Our results indicate that anchoring of the K1237 side chain via an inter-domain link is necessary in order to obtain a consistent description of the Na^+^/K^+^ selectivity.

## Conclusions

We have investigated ion permeation properties of a Na_V_1.4 homology model, which was previously validated using the mutagenesis data for binding of *μ*-conotoxins. A novel feature of the Na_V_1.4 model is the inter-domain links involving the side chains of DEKA residues, which could otherwise block the permeation pathway in the SF. Unbiased MD simulations and PMF calculations reveal that only one Na^+^ ion can bind to the SF while the vestibule can be occupied by more than one Na^+^ ion, partly coordinated by the side chains of the EEDD residues. The role of the EEDD ring appears to be to concentrate Na^+^ ions in the vestibule without binding them to any particular site. Ion permeation is based on a simple knock-on mechanism, where a Na^+^ ion in the vestibule goes over the free energy barrier at the entry of the SF and pushes the Na^+^ ion in the SF to the intracellular cavity. This is much simpler than the permeation process in bacterial Na_V_ channels, which involves three Na^+^ ions moving through a complicated free energy landscape in the SF via a knock-on or a pass-by mechanism [23, 24]. Thus, unlike in potassium channels, the lessons learned from the bacterial Na_V_ channels about the permeation mechanism do not appear to be directly transferable to the mammalian Na_V_ channels.

Using the Na_V_1.4 model, we have also successfully reproduced the Na^+^/K^+^ selectivity properties, which provides further validation of the proposed model. Thus, overall the model provides a reasonable platform for future studies of ion permeation and ligand binding in mammalian Na_V_ channels. More quantitative characterization of the permeation properties of the Na_V_1.4 model is needed to fully validate it. To this end, construction of an open state of the model channel would be very valuable.

The PDB coordinates of the Na_V_1.4 model are available from the authors upon request.
